# Cyclic Fatigue Resistance of Heat-Treated Nickel-Titanium Instruments

**DOI:** 10.22037/iej.v13i3.18637

**Published:** 2018

**Authors:** Mário Tanomaru-Filho, Camila Galletti Espir, Ana Carolina Venção, Nathaly Macedo-Serrano, Jader Camilo-Pinto, JulianeMaria Guerreiro-Tanomaru

**Affiliations:** a * Department of Restorative Dentistry, Araraquara School of Dentistry, Univ Estadual Paulista, Rua Humaitá, 1680, Centro, Araraquara, São Paulo, Brazil*

**Keywords:** Cyclic Fatigue, Instrumentation, Nickel-Titanium, Rotary System

## Abstract

**Introduction:**

This study compared the cyclic fatigue resistance (CFR) of new instruments manufactured by heat-treated nickel-titanium wire.

**Methods and Materials:**

Ninety-six new instruments from HyFlex CM (HF), Edge File (EF), Pro Design S (PDS/L) and Mtwo (MT) (20/0.06 and 25/0.06) (*n*=12) systems were evaluated. A stainless steel device was used and time and number of cycles to fracture (NCF) were observed. Fragments were measured and fracture surface was evaluated using scanning electron microscope (SEM). ANOVA and Tukey’s tests were applied with the level of significance set at 0.05.

**Results:**

PDS 20/0.06 and PDL 25/0.06 instruments presented the highest CFR. MT 20/0.06 and MT 25/0.06 showed the lowest CFR (*P*<0.05). The length of fragments was similar for 25/0.06 instruments and HF presented the highest one for 20/0.06 instruments. SEM analyses showed morphology suggestive of ductile fracture.

**Conclusion:**

Heat treatment increased resistance to cyclic fatigue differently for each type of instrument. PDS 20/0.06 and PDL 25/0.06 present higher cyclic fatigue resistance.

## Introduction

Advances in the manufacturing of rotary Nickel-Titanium (NiTi) instruments provide a better perspective to endodontic treatment increasing resistance to fatigue [1-3]. Resistance to cyclic fatigue corresponds to the number of cycles an instrument can withstands under repeated cycles of tension and compression, until fracture and the dispersion of the lifetime of NiTi instruments, and their deflecting load (DL) changes during cyclic fatigue [4]. The thermodynamic properties of NiTi alloys involving memory shape heat treatment produced instruments significantly more resistant to fatigue [5] and also improved flexibility [3]. Furthermore, studies have demonstrated the superiority of thermally treated instruments related to fatigue resistance [5-7].

The HyFlex CM (Coltene/Whaledent AG, Altstatten, Switzerland) is a NiTi System with controlled memory instruments manufactured by a thermal process, which through a specific heating and cooling process, allows for a 300% increase in flexural strength in conventional NiTi alloys [8, 9].

The ProDesign instruments (Easy Dental Equipment, Belo Horizonte, Brazil) are manufactured with NiTi alloy with heat treatment CM, similar to the treatment applied to the HyFlex CM instruments. This treatment modifies the phase transformation of the NiTi alloy, making it martensitic at room temperature, increasing its flexibility and flexural strength [10]. The ProDesign Logic instruments (Easy Dental Equipment, Belo Horizonte, Brazil) follow the concept of single-file, associated with heat treatment CM, with s-shaped cross-section [11]. Removal of the filling material after the use of alternating and rotating movements in curved canals showed that the ProDesign Logic 50/0.01 file for apical preparation reduced the amount of material remaining in the apical portion [12].

The Edge File system (EdgeEndo, EDGEFILE, Albuquerque, New Mexico, USA) has instruments manufactured by a heat treatment process (FireWire NiTi), which according to the manufacturer increases the flexural strength and flexibility of NiTi instruments [13]. The EdgeFile instruments obtained greater resistance to cyclic fatigue than the Vortex Blue and ESX instruments at three temperatures evaluated at 3^°^C, 22^°^C, 37^°^C, and 60^°^C [14]. 

The aim of this study was to evaluate the cyclic fatigue resistance of heat-treated instruments size 20/0.06 taper from HyFlex CM (Coltene –Whaledent, Altstatten, Switzerland), Edge File (Edge Endo, EDGEFILE®, Canada), Pro Design S (Easy, Belo Horizonte, Brazil) and MTwo (VDW, Munich, Germany) systems and size 25/0.06 taper from HyFlex CM, Edge File, Pro Design Logic and MTwo. The null hypothesis is that the heat treatment of the instruments do not improve the fatigue resistance, with no difference among the instruments.

## Materials and Methods

Ninety-six instruments with different heat treatment (CM-Wire and Fire-Wire) and without heat treatment were evaluated according to Table 1. Each instrument was inspected regarding the presence of defects or deformities before the experiment under a stereomicroscope (Carl Zeiss, LLC, Oberkochen, Germany) under ×16 magnification. The static bending test were performed in a device simulating an artificial canal with a 45^°^ angle and 5-mm radius of curvature [15]. 

***Testing device***

The device used for the tests consisted of an iron base (50×30 cm) with a fixing support for the low speed handpiece (Dabi Atlante S/A Indústrias Médico Odontológicas, Ribeirão Preto, SP, Brazil). After the instruments had been coupled to the low-speed handpiece, the height of the support was adjusted so that the instrument could be inserted into the artificial canal, without causing stress and allowing free rotation. The curvature of the stainless-steel artificial canal was fitted onto a cylinder guide made of the same material. During the tests, the instruments were placed with 1 mm of the instrument tip protruded beyond the end of the artificial canal walls, allowing visualization of the tip and determination of moment of instrument fracture. 

**Table 1:** Manufacturer and production process of evaluated instruments

**Instrument (** ***n*** **=12)**	**Manufacturer**	**Production process**
**HyFlex CM 20/0.06**	Coltene, Altstatten, Switzerland	CM heat treatment
**HyFlex CM 25/0.06**	Coltene, Altstatten, Switzerland	CM heat treatment
**EdgeFile X7 20/0.06**	EdgeFile, EDGEFILE®, Canada	FireWire NiTi
**EdgeFile X7 25/0.06**	EdgeFile, EDGEFILE®, Canada	FireWire NiTi
**ProDesign S 20/0.06**	Easy, Belo Horizonte, Brazil	CM heat treatment
**ProDesign Logic 25/0.06**	Easy, Belo Horizonte, Brazil	CM heat treatment
**MTwo 20/0.06**	VDW, Munich, Germany	Conventional NiTi
**MTwo 25/0.06**	VDW, Munich, Germany	Conventional NiTi

**Table 2:** Mean (SD) of the number of cycles to fracture, time up to instrument fracture and length (in mm) of fragments (*n*=12) to 20.06 instruments

**Groups (** ***n*** **=12)**				
	**HyFlex 20/0.06**	**Edge File 20/0.06**	**PDS 20/0.06**	**MTwo 20/0.06**
**Number of cycles to fracture**	2446 (513.7)^b^	1500 (305.6)^c^	4370 (928.4)^a^	853.7 (68.13)^d^
**Time up to fracture**	8.15 (1.71)^b^	5.0 (1.02)^c^	14,57 (3.09)^a^	2,842 (0.22)^d^
**Fragment lengths**	6.006 (0.61)^a^	4.88 (0.54)^c^	5,250 (0.59)^b,c^	5,575 (0.64)^a,b^

* *Equal letters on the same line indicate statistical similarity (P>0.05)*

**Table 3:** Mean (SD) of the number of cycles to fracture, time up to instrument fracture and length (in mm) of fragments to 25/0.06 instruments

**Groups ** **(** ***n*** **=12)**				
	**HyFlex 25/0.06**	**Edge File 25/0.06**	**Pro Desing Logic 25/0.06**	**MTwo 25/0.06**
**Number of cycles to fracture**	2195 (479,6)^b^	1030 (213.0)^b^	9481 (2664)^a^	854,2 (70,51)^b^
**Time up to fracture**	7.32 (1,525)^b^	3.43 (0.709)^b^	31.60 (8.881)^a^	2,825 (0,273)^b^
**Fragment lengths**	6.286 (0.535)^a^	6.237 (1.178)^a^	7.196 (1.216)^a^	6,290 (0,462)^a^

* *Equal letters on the same line indicate statistical similarity (P>0.05)*

**Figure 1:**
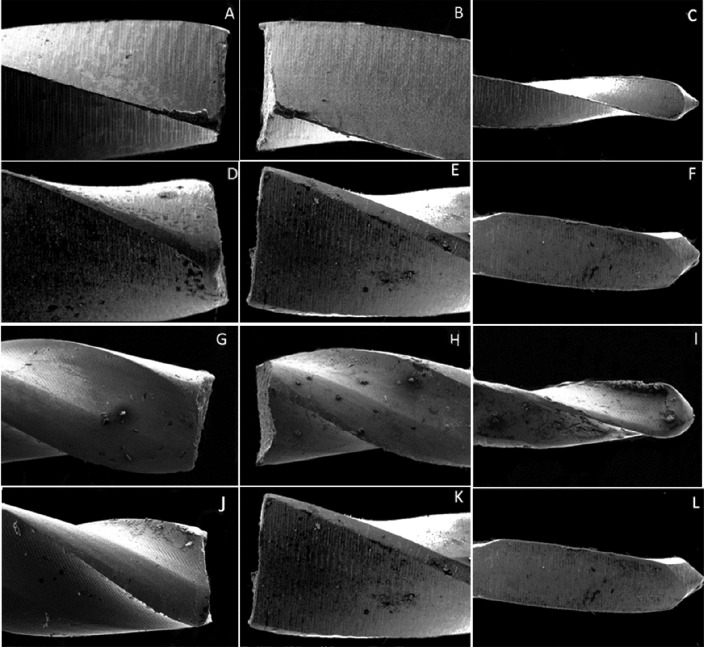
Electron microscopy micrographs of fractured instruments in lateral view: Hyflex 20/0.06 *(A, B, C)*; Hyflex 25/0.06 *(D, E, F);* EdgeFile 20/0.06 *(G, H, I)*; EdgeFile 25/0.06 *(J, K, L),* under ×150 magnification; Characteristics of ductile flexural fracture were observed

The instruments were used at 300 rpm for standardization between systems, since this speed is compatible to the safety standards of all the instruments presented and a torque of 250 g/cm in continuous rotary motion, driven with the electric motor VDW Silver (VDW Silver, GmbH, Munich, Germany). All instruments were rotated until fracture and WD-40 synthetic oil (WD-40 Multipurpose Product, Brazil) was used to reduce friction. The number of cycles to failure (NCF) was obtained by multiplying the time (in min) to failure by the number of rotations or cycles per min (300 rpm). The length of fractured instrument fragments was measured by using a digital caliper (Digimess, São Paulo, SP, Brazil).

***Scanning electron microscopy (SEM)***

To perform this analysis, five instruments from each group were randomly selected. The fractured instruments and fragments were cleaned using a detergent solution in an ultrasound bath for 120 sec before examination with the SEM. The fracture surfaces of the instruments and fragments were examined under a scanning electron microscope (JEOL, JSM–6610LV Scanning Electron Microscope, Peabody, MA, USA). The fragments were examined in a lateral view with a magnification of ×150, followed by a fractographic examination, with the fracture end facing upward [16].

***Statistical analysis***

Data were first verified regarding normality of the distribution and then analyzed by using ANOVA and post-hoc Tukey’s tests, with the significance level set at 0.05.

**Figure 2:**
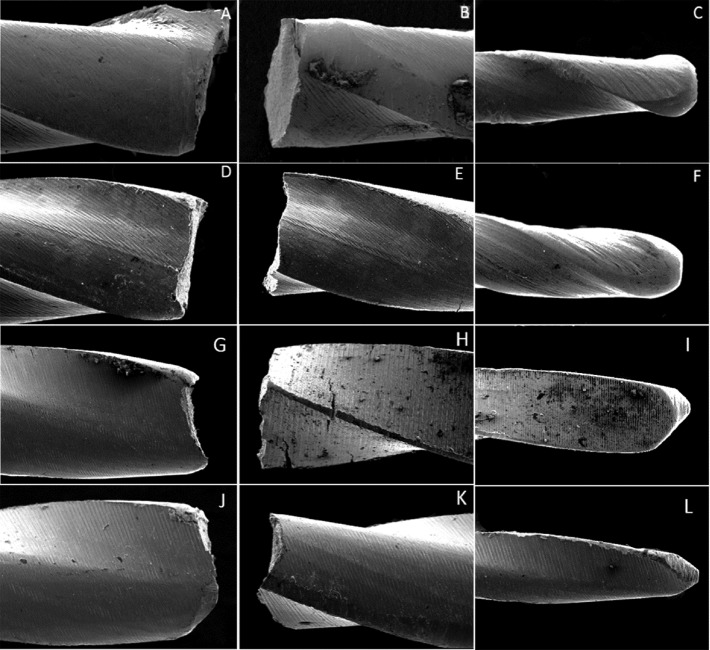
Electron microscopy micrographs of fractured instruments in lateral view: Pro Design S *(A, B, C)* 20/0.06; Pro Design Logic *(D, E, F)* 25/0.06; MTwo 20/0.06 *(G, H, I)*; and MTwo 25/0.06 *(J, K, L)*, under ×150 magnification. Characteristics of ductile flexural fracture were observed

## Results

The mean and standard deviations of the cyclic fatigue resistance, the time up to fracture and the length of fragments are presented in Tables 2 and 3. NCF and time up to fracture of size 20/0.06 instruments were significantly higher for Pro Design S (*P*<0.05). In the comparison of size 25/0.06 taper instruments, there were no significant differences in the NCF and time up to fracture (*P*>0.05) between MTwo and EdgeFile, as well as HyFlex and Edge File systems. ProDesign Logic instruments presented a higher value (*P*<0.05). There was no difference in NFC and time to fracture between the HyFlex, Edge File and MTwo instruments of size 25/0.06 (*P*>0.05). The ProDesign Logic 25/0.06 instruments obtained longer time in cyclic fatigue and higher NFC.

**Figure 3:**
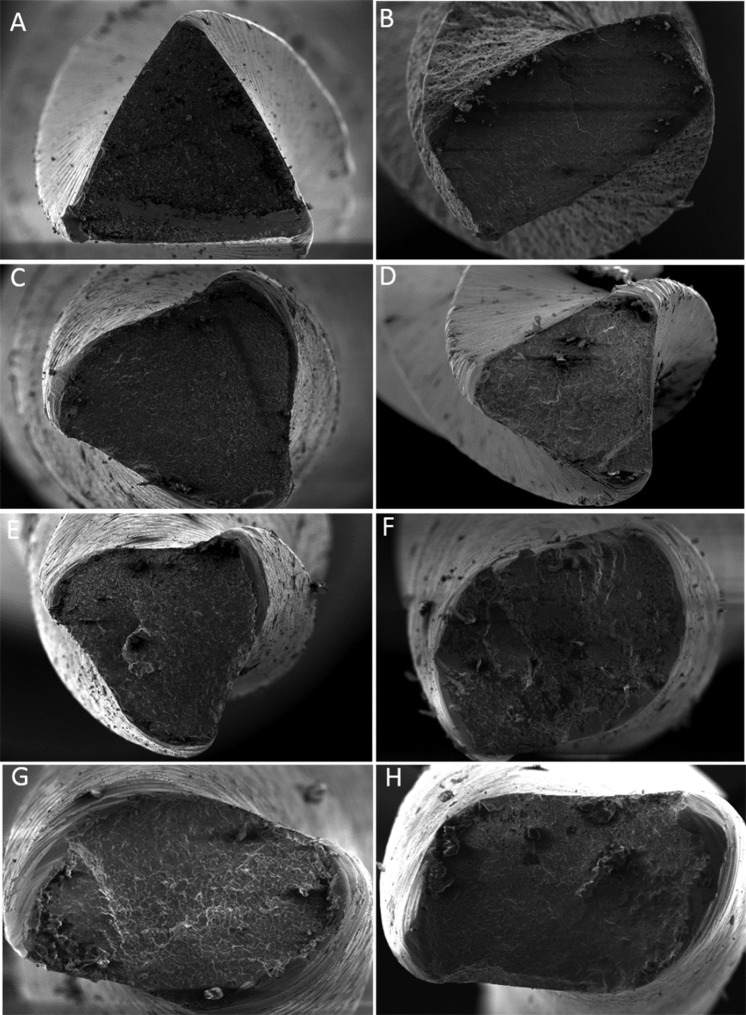
Scanning electron micrographs of the fracture surface after cyclic fatigue test. HyFlex 20/0.06 and 25/0.06 (*A and B*), EdgeFile 20/0.06 and 25/0.06 (*C and* *D*), ProDesign 20/0.06 and 25/0.06 (*E and F*) and Mtwo 20/0.06 and 25/0.06 (*G and H*

No statistically significant difference was found between the level of fragments for size 25/0.06 taper instruments. The level of fragments had no statistically significant difference for size 25/0.06 taper instruments (*P*>0.05), with significant differences between HyFlex and Edge File and HyFlex and Pro Design S to size 20/0.06 taper instruments (*P*<0.05). SEM analyses revealed that the instruments exhibited morphological characteristics suggestive of ductile fracture on the fractured surfaces. The surface features of each brand of instrument under the SEM are shown in Figures 1, 2 and 3. No evidence of plastic deformation was observed on the helical axes (Figure 3).

## Discussion

Fatigue resistance is widely evaluated using this type of device [15, 17-21]. The standardized artificial canals with different lengths, degree and radius of curvatures and diameters has been used in several studies [19-26] and the improvement of this methodology has also been analyzed [27]. In the present study, the device simulated a root canal with 45^°^ angle and 5-mm radius of curvature, as previously described. In the present study, a device simulated root canal, of stainless steel was used, with a 45^°^ angle and 5 mm radius of curvature, as described in previous studies [15, 17]. In the present study The stainless steel device was used to simulate a root canal with the angled and 45^°^ and 5 mm radius, with the intuition to evaluate the flexural strength in the 4 mm closest to the tip of the instruments, since this is the region in which fractures of NiTi are more frequently occurring. Severe curvatures are classified with values from 25 to 70^°^ [28]. 

The development of alloys and treatments are related to better cyclic fatigue. Heat treatment contributes to increase austenite transformation temperature, with significant changes in the phase transformation behavior, compared with conventional superelastic NiTi instruments [29]. In the present study, different manufacturing process and design were chosen to evaluate cyclic fatigue resistance. This study compared Pro Design and HyFlex instruments, which are manufactured using CM heat treatment; Edge File instruments, which are manufactured by a heat treatment process (FireWire NiTi); and MTwo instruments, which have a traditional manufacturing process with no thermal treatment. 

The ProDesign systems obtained greater resistance to cyclic fatigue than the other instruments (HyFlex CM, EdgeFile and MTwo) both in the comparison between instruments 20/0.06 and 25/0.06. HyFlex 20/0.06 got the second position, losing in flexural strength for ProDesign instruments and getting better results than MTwo and Edge File. The heart treatment CM that is applied to the ProDesign and HyFlex CM systems, contributed positively to the best results presented in relation to instruments without heat treatment (MTwo) or with Fire Wire heart treatment (Edge File). The CM treatment is obtained by heating and cooling the instruments after their machining. This procedure modifies the microstructure of the instruments, changing their behavior of phase transformation, thus the instrument presents the martensitic phase in clinical situation. Instruments in the martensitic form are more malleable, soft and ductile and can be easily deformed, different from austenitic Nickel and Titanium that is stronger and harder in comparison to martensitic [29]. The martensitic alloy has a geminated structure, which causes greater damping and absorption of energy, thus causing the crack zones to be multiple and more gradual, thus delaying their complete fracture [3].

The ProDesign Logic systems obtained better results compared to the HyFlex CM system even the two systems presenting the same CM heat treatment. This difference can be explained appear to be due to the different cross sections between the instruments. The cross-sectional shape of an instrument has a significant impact on bending stresses [30]. The S format of the ProDesign instruments may have contributed to their better flexural strength since instruments with two cutting edges tend to have a lower mass and subsequently be more flexible and with greater resistance to cyclic fatigue [31].

In the comparison of size 20/0.06 taper instruments, heat-treated instruments had greater cyclic fatigue resistance than MTwo (no thermal treatment). Specific thermal treatments promote proper results in cyclic fatigue resistance of the NiTi instruments [29]. Heat treatment or thermal processing adjusts the transition temperature in NiTi alloy, improving the flexibility and fatigue resistance of NiTi endodontic files [32]. Instruments made from conventional NiTi alloys exhibit an austenitic phase at room temperature during clinical applications [33], presenting characteristics of strength and hardness. M-Wire and CM instruments, in addition to the austenite, also contained martensitic B190 and R phase, associated with soft and ductile characteristics, and also shape memory effect and superelasticity [3, 33]. Besides that, the lowest mean of NCF to MTwo system can be attributed to the traditional grinding process. This process of production, besides influencing fatigue resistance [34, 35], forms microcracks and defects within the internal structure and along the surface of the files, which results in points of stress concentration. Consequently, cracks can propagate to failure at a stress level lower than the stress typically experienced during canal instrumentation and result in unexpected file fracture [24]. SEM observations of the surface characteristics and features of the instruments (Figure 3) revealed that after fatigue testing, the majority of specimens showed an increased amount of surface microcracks (in lateral view) near the location of fracture.

In the comparison of size 25/0.06 instruments, Edge File (files with heat-treatment) had similar cyclic fatigue resistance to MTwo (files with no heat-treatment) system, and significantly less cyclic fatigue resistance to Pro Design Logic. In another study, HyFlex system (files with heat-treatment) showed similar results to ProTaper Next X2 (M-Wire) in the cyclic fatigue resistance in the apical curvature of an artificial canal with a double curvature [6]. These results show that heat treatment increased resistance to cyclic fatigue differently for each type of instrument. Besides that, in the comparison of smaller diameter instruments (20/0.06), thermal treatments improved the resistance of the files, with better results to heat-treated files. The dimensions of the instruments can influence its cyclic fatigue resistance and this property decreased as the instrument diameter increased [36]. A progressive reduction in flexibility may also occur with increase in the diameter and taper of the instrument [36]. Furthermore, varied cross-sections may also interfere in the resistance of the instrument [5] and instruments with larger cross-sectional areas present greater torsional rigidity and consequently lower resistance to fatigue [17]. The material properties, design and dimensions of each instrument are specific to each brand tested and cannot be totally eliminated during the test, making it difficult to quantify the effect of a single variable on fatigue behavior [37].

## Conclusion

Therefore, it is possible to conclude that heat treatment increased resistance to cyclic fatigue differently for each type of instrument. ProDesign S 20/0.06 and ProDesign Logic 25/0.06 instruments presented higher cyclic fatigue resistance values when compared with HyFlex and Edge File systems.
